# Pathogenic variants in *PIDD1* lead to an autosomal recessive neurodevelopmental disorder with pachygyria and psychiatric features

**DOI:** 10.1038/s41431-021-00910-0

**Published:** 2021-06-24

**Authors:** Maha S. Zaki, Andrea Accogli, Ghayda Mirzaa, Fatima Rahman, Hiba Mohammed, Gloria Liliana Porras-Hurtado, Stephanie Efthymiou, Shazia Maqbool, Anju Shukla, John B. Vincent, Abrar Hussain, Asif Mir, Christian Beetz, Anika Leubauer, Henry Houlden, Joseph G. Gleeson, Reza Maroofian

**Affiliations:** 1 Clinical Genetics Department, Human Genetics and Genome Research Division, National Research Centre, Cairo, Egypt; 2 The Department of Medical Genetics, McGill University Health Centre, Montreal, QC Canada; 3 Center for Integrative Brain Research, Seattle Children’s Research Institute, Seattle, WA USA; 4 Development and Behavioural Pediatrics Department, Institute of Child Health and The Children Hospital, Lahore, Pakistan; 5 American centre for Psychiatry and Neurology’, Abu Dhabi, United Arab Emirates; 6 Department of Medical Genetics, INCERHC International center research health Comfamiliar - Salud Comfamiliar, Comfamiiar Risaralda, Pereira, Colombia; 7 Department of Neuromuscular Diseases, UCL Queen Square Institute of Neurology, Queen Square, WC1N3BG London, London, UK; 8 Department of Medical Genetics, Kasturba Medical College, Manipal, Manipal Academy of Higher Education, Manipal, Karnataka India; 9 Molecular Neuropsychiatry & Development (MiND) Lab, Campbell Family Mental Health, Research Institute, Centre for Addiction and Mental Health, Toronto, ON Canada; 10 Institute of Medical Science, University of Toronto, Toronto, Ontario Canada; 11 Human Molecular Genetics Lab, Department of Biological Sciences, FBAS, International Islamic University, Islamabad, Pakistan; 12 CENTOGENE AG, Rostock, Germany; 13 Department of Neurosciences, University of California, San Diego, CA USA; 14 Rady Children’s Institute for Genomic Medicine, San Diego, CA USA

**Keywords:** Clinical genetics, Medical research

## Abstract

The PIDDosome is a multiprotein complex, composed by the p53-induced death domain protein 1 (PIDD1), the bipartite linker protein CRADD (also known as RAIDD) and the proform of caspase-2 that induces apoptosis in response to DNA damage. In the recent years, biallelic pathogenic variants in *CRADD* have been associated with a neurodevelopmental disorder (MRT34; MIM 614499) characterized by pachygyria with a predominant anterior gradient, megalencephaly, epilepsy and intellectual disability. More recently, biallelic pathogenic variants in *PIDD1* have been described in a few families with apparently nonsydnromic intellectual disability. Here, we aim to delineate the genetic and radio-clinical features of PIDD1-related disorder. Exome sequencing was carried out in six consanguineous families. Thorough clinical and neuroradiological evaluation was performed for all the affected individuals as well as reviewing all the data from previously reported cases. We identified five distinct *novel* homozygous variants (c.2584C>T p.(Arg862Trp), c.1340G>A p.(Trp447*), c.2116_2120del p.(Val706Hisfs*30), c.1564_1565delCA p.(Gln522fs*44), and c.1804_1805del p.(Gly602fs*26) in eleven subjects displaying intellectual disability, behaviorial and psychiatric features, and a typical anterior-predominant pachygyria, remarkably resembling the CRADD-related neuroimaging pattern. In summary, we outlin`e the phenotypic and molecular spectrum of PIDD1 biallelic variants supporting the evidence that the PIDD1/CRADD/caspase-2 signaling is crucial for normal gyration of the developing human neocortex as well as cognition and behavior.

## Introduction

The PIDDosome is a multiprotein complex that drives activation of the endopeptidase caspase-2 upon genotoxic stress to induce apoptosis [1, 2]. It is composed by the p53-induced death domain protein 1 (PIDD1), the bipartite linker protein CRADD (also known as RAIDD) and the proform of caspase-2. The formation and activation of the PIDDosome complex is a dynamic and tightly regulated process that follows a cascade of events requiring as a first critical step the interaction of CRADD and PIDD1 through their death domains (DD) to further recruit caspase-2 that determines cell death initiation [3].

In the last few years, biallelic pathogenic variants in *CRADD* have been associated with a neurodevelopmental disorder (MRT34; MIM 614499) described as “thin” lissencephaly (TLIS) variant and characterized by pachygyria with a predominant anterior gradient, megalencephaly, epilepsy and intellectual disability (ID) [4]. This finding has raised attention on the PIDDosome complex, revealing additional biological functions aside the DNA-damage induced apoptosis [5]. In this regard, the CRADD-interacting protein PIDD1 can function as a sensor surveilling centrosome numbers, critically regulating cellular differentiation processes during organogenesis and regeneration [6, 7].

Recently, 4 homozygous variants in *PIDD1* have been reported in 11 subjects across 5 different families with nonsydnromic ID with scarce clinical and neuroimaging details [8–10].

Here, we present 11 new subjects from 6 different families harboring 5 *novel* homozygous variants in *PIDD1* and thoroughly review radio-clinical data from previously reported patients, outlining the molecular and phenotypic spectrum of *PIDD1*-related disorder.

## Material and methods

### Patients and genetic analysis

**Fig. 1:**
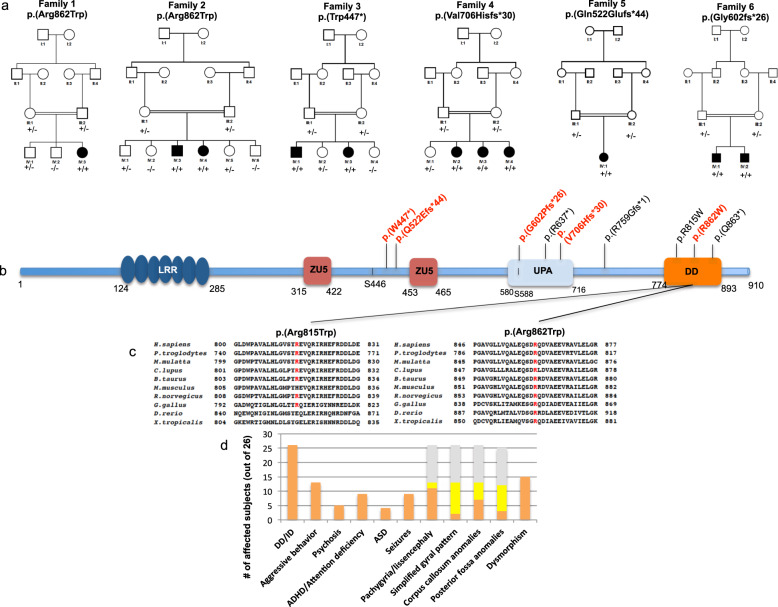
Pedigree of the families, localization and conservation of PIDD1 variants. **a** Pedigree of families 1–6. In the pedigree, squares represent males, circles females, black shaded symbols denote affected patients harboring biallelic PIDD1 variants; gray shaded symbol refers to a subject with two episodes of generalized motor seizure. Plus (+) and minus (−) signs indicate presence or absence of the PIDD1 variants ([+/+] homozygote, [+/−] heterozygote and [−/−] wild-type). **b** Schematic depiction of the full length PIDD1 shows seven leucine-rich repeats (LRR; dark blue), two ZU5 domains (present in ZO-1 and Unc5-like netrin receptors; purple), an UPA domain (conserved in UNC5, PIDD1 and ankyrins; blue light), and the C-terminal death domain (DD; orange). Autoprocessing of full-length PIDD1 at positions S446 and S588 can give rise to three protein fragments that contain different structural motifs relevant for protein-protein interaction and complex formation. Variants identified in the current cohort are displayed in bold and red. **c** The HomoloGene-generated amino acid aligment of human PIDD1 and its predicted orthologs shows the conservation of the amino acid R815 and R862. **d** Bar graph showing the distribution of the most relevant clinical features among the total subjects (26) identified so far with biallelic PIDD1 variants. (Orange: number of patients out of 26 showing each feature. Yellow: number of patients without each specific feature. Gray: brain MRI not available for 13 subjects).

Eleven previously unreported subjects from six unrelated families of different ancestries (Egyptians, Pakistani, Palestinian and Colombian) where included in this study after written informed consent was obtained from the parents (Fig. 1a, Supplementary Table 2).

Detailed clinical presentations and family history were recorded for all the subjects. Brain magnetic resonance imaging (MRI) findings were reviewed by a pediatrician expert in malformations of cortical development (GM). We also carefully reviewed brain MRI of 2 cases previously reported [8, 10], provided follow-up data of previously described cases and included clinical details of 4 additional individuals of the family AS110 previously described [8]. Exome sequencing (ES) was performed in probands in 4 centers as described previously [11, 12]. Sanger sequencing with standard methods was performed for candidate variants validation and familial segregation. All *PIDD1* variants are reported according to the NM_145886.4 transcript and classified according to the ACMG criteria.

## Results

### Genetic findings

ES revealed 5 *novel* or ultra-rare PIDD1 variants in homozygous status as follow: the homozygous missense variant NM_145886.4: c.2584C>T p.(Arg862Trp) in subject IV:3 of Family 1 and two siblings of family 2 (IV:3, IV:4), the homozygous nonsense variant c.1340G>A p.(Trp447*) in subject IV:1 and IV:3 of family 3, the frameshift variant c.2116_2120del p.(Val706Hisfs*30) in the siblings IV:2, IV:3, IV:4 of family 4; the homozygous frameshift variant c.1564_1565del p.(Gln522fs*44) in patient IV:1 of family 5; the homozygous frameshift variant c.1804_1805del, p.(Gly602fs*26) in subjects IV:1 and IV:2 of family 6. Sanger Sequencing confirmed segregation of the variants with the phenotype within these families.

All variants are extremely rare in human population variant databases (allele frequency ranging from 0 to 0.00003285 in gnomAD), never reported in homozygous state in healthy individuals (Supplementary Table 1). The frameshift variants c.2116_2120del p.(Val706Hisfs*30), c.1564_1565del p.(Gln522fs*44) and c.1804_1805del p.(Gly602fs*26) and the nonsense variants c.1340G>A p.(Trp447*) are predicted to result in a premature truncation of the transcript, likely leading to nonsense-mediated mRNA decay. The missense variant c.2584C>T p.(Arg862Trp), found in two unrelated Egyptian families, affects a highly conserved residue (GERP 4.74, CADD 31) (Fig. 1d) and is predicted to have a deleterious effect by *in-silico* analysis (Supplementary Table 1). This variant is most likely a founder variant as the families come from the same region in Egypt. Additionally, no other pathogenic/likely pathogenic variants were identified in the currently known NDD-related genes in the ES data in these families.

### Clinical and neuroradiological characteristics of the patients

Our cohort consists of 11 affected children from 6 different consanguineous families (mean age 13.2 years, range 23.4). All subjects had developmental delay and variable degree of ID (mild = 4, moderate = 6). None of them had developmental regression. Six and four subjects met diagnostic criteria for Attention deficit/hyperactivity disorder (ADHD) and Autism spectrum disorder (ASD), respectively. All but one subjects had aggressive and self-mutilation behaviors, such as head banging and hand biting. Two individuals developed psychosis presenting with delusions and hallucinations that required antipsychotic treatment. Three subjects experienced generalized motor seizures and one focal motor seizure that were well controlled by anti-seizure medications. Apart from mild hypotonia, strabismus and gait instability in a few subjects there were no major neurological deficits at the neurological exam. Mild and non-specific dysmorphic features were noticed in the majority of subjects, being prominent forehead the most common feature (Supplementary Fig. 1).

**Fig. 2:**
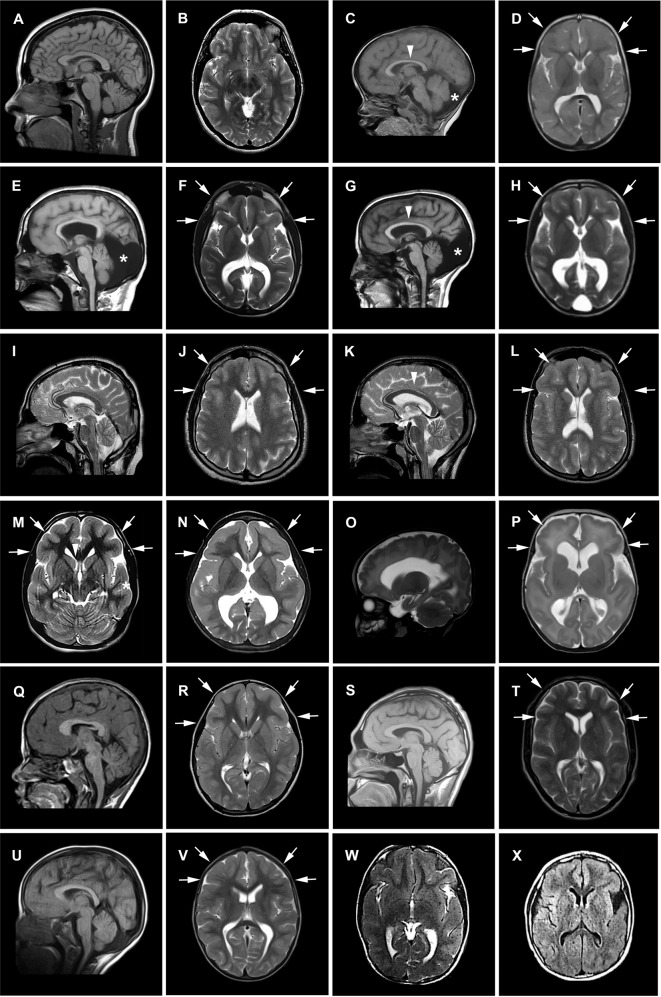
Neuroradiological features of patients with PIDD1 variants. Representative mid-sagittal and axial brain MR images are shown. **A**, **B** Mid-sagittal and axial images of an unaffected individual (normal). **C**, **D** Images of subject IV:3 of family 1 showing anterior-predominant pachygyria (arrows), thin corpus callosum (arrowhead), mild cerebellar vermian hypoplasia with a retrocerebellar cyst (asterisk). Images of family 2 (**E**, **F** G-IV:3; **G**, **H** IV:4) showing severe anterior-predominant undersulcation of the cortical gyral pattern consistent with pachygyria and foreshortening of the frontal lobes (arrows), thin corpus callosum, and retrocerebellar cyst (or mega-cisterna magna) (asterisk). Images of family 3 (**I**, **J** IV:1; **K**, **L** IV:3) showing frontal-predominant undersulcation of the cortical gyral pattern, with no cerebellar or callosal abnormalities (arrows). Axial images of family 4 (**M** IV:2; **N** IV:3) showing frontal-predominant pachygyria with foreshortening of the frontal lobes (arrows). IV:3 also has ventriculomegaly (limited views). **O**, **P** Images of subject IV:1 of family 5 showing severe undersulcation and pachygyria diffuse (more pronounced frontally; arrows) with ventriculomegaly. **Q**, **R** Images of family Manipal-1 (IV:3) (previously described) showing anterior-predominant pachygyria (arrows) and thick genu of the corpus callosum (arrowhead). **S**, **T** Images of affected subjects of families AS110 V-3 (previously described) showing undersulcation of the frontal, temporal and parietal regions resembling lissencephaly (arrows). **U**, **V** AS105 III-5 (previously described) showing frontal pachygyria with mildly to moderately thick cortex and mild hypoplasia of the corpus callosum. **W**, **X** axial images of family 6 showing diffuse undersulcation of the cortical gyral pattern with a thick appearing cortex on limited views.

Brain MRI, available for 10 of 11 subjects revealed cortical anomalies in all, mainly consistent with a predominant anterior pachygyria in eight and dysgyria/simplified gyral pattern of the frontal lobes in two. Specifically, the thickness of the cortex was above the normal limit of 4 mm for most cortical regions in patients with the pachygyria appearance on brain MRI. Half of the cohort had corpus callosum anomalies, mostly thin corpus callosum. Posterior fossa anomalies including retrocerebellar cyst and megacisterna magna were observed in three subjects along with vermis hypoplasia in one of them. Two individuals also had lateral ventricle enlargement. In addition, re-reviewing the brain MRI of 2 previously reported cases [8, 10] was reminiscent of the pattern seen in our patients, unveiling the presence of mildly thick cortical gyral pattern also in one of them, thought to have only mildly corpus callosum hypoplasia (Fig. 2). Clinical features are summarized in Table 1 and Fig. 1d and extendedly reported in more details in Supplementary Table 2 that also include data of the previously described cohorts.

**Table 1:** Genetic and phenotypic features of subjects with PIDD1 variants.

	Family 1	Family 2		Family 3		Family 4				Family 5	Family 6		Sheikh et al. 2021	Harripaul et al. 2018		Hu et al. 2019	
Subject	G-3771 IV:1	G-4482 IV:3	G-4482 IV:4	IV:1	IV:3	IV:2	IV:3	IV:4		IV:2	IV:1	IV:2	Manipal-1 IV:3	[7] subjects Family AS110^b^	[2] subjects Family AS105	[2] subjects Family M278	[3] subjects Family M8700004
PIDD1 variant (NM_145886.4)	c.2584C>T p.(Arg862Trp)	c.2584C>T p.(Arg862Trp)		c.1340G>A p.(Trp447^a^)		c.2116_2120del p.(Val706Hisfs^a^30)				c.1564_1565del p.(Gln522fs^a^44)	c.1804_1805del p.(Gly602fs^a^26)		c.1909C>T p.(Arg637^a^)	c.2587C>T p.(Gln863^a^)	c.2587C>T p.(Gln863^a^)	c.2275-1G>A p.(Arg759Gly^a^1)	c.2443C>T p.(Arg815Trp)
Zigosity	hmz	hmz		hmz		hmz				hmz	hmz		hmz	hmz	hmz	hmz	hmz
Ethnicity	Egyptian	Egyptian		Egyptian		Pakistani				Palestinian	Colombian		Indian	Pakistani	Pakistani	Iran	Iran
Age, sex	8 y, F	22 y, M	18 y, F	24 y, M	17 y, F	14 y, F	11 y, F		8 y, F	8 m, F	11 y, M	15 y, M	10 y, F				
DD/ID	Mild	Mode-rate	Mild	Mode-rate	Mode-rate	Mode-rate	Mode-rate		Mode-rate	DD^a^	Mild	Mid	Mild	Mild-moderate [7]	Severe [2]	Moderate [2]	Severe [3]
ASD	+	+	−	+	+	−	−		−	^a^	−	−	−	−	−	−	−
ADHD	+	+	−	+	+	−	−		−	^a^	+	+	+	+ (1)	+	−	−
Aggressive behavior	Head banging	Hitting his parents, biting his hands	+	Hitting his parents	+	Self-mutilation	Self-mutilation		Self-mutilation	^a^	−	+	−	+ (2)	+ (2)	−	−
Other behavior –Psychiatric issues	Psychosis, masturbation-like-movement	Coprolalia, tantrums	Psychosis, coprolalia	Tantrums	Tantrums	−	−		−	−	−	−	Nail biting	+ (2)	+ (1)	−	Psychosis(3)
Seizures	−	+	+	+	−	−	−		−	−	−	+	+	+ (1)	−	−	+ (3)
Brain MRI findings	A-P pachygyria, thinning CC, vermian hypoplasia retrocerebellar cyst	A-P pachygyria, thinning CC, MCM	A-P pachygyria, thinning CC, MCM	Dysgyria/ simplifiedfrontal cortex	Dysgyria/simplified frontal cortex	Frontal pachygyria	A-P pachygyria		NA	A-P pachygyria/ lissencephaly, thinning CC, ventriculomegaly	A-P pachygyria, mildly thinning CC	A-P pachygyria	A-P pachygyria, thick genu of CC	Lissencephaly	Frontal pachygyria, mild thinning CC (1)	NA	NA
Neurological exam	Strabismus hypotonia, brisk DTR, mild gait instability	Strabismus, Brisk DTR, mild tremor	Brisk DTR	Brisk DTR	Brisk DTR, mild hyotonia	Gait instability	Normal		Gait imbalance	Axial hypotonia	Strabismus	Axial hypotonia	Normal	Bradychynesia (2)	Strabismus (2)	Normal	Normal
Dysmor-phism	+	+	+	+	+	+	+		+	+	+	+	+	−	+ (1)	+ (2)	−

A-P anterior-posterior, CC corpus callosum, DD developmental delay, DTR deep tendon reflexes, F female, hmz homozygous, ID intellectual disability, y years, m months, M male, NA not available.

^a^Too young to be assessed.

^b^Four of the seven subjects are new patients described in the current study.

## Discussion

In the present study, we report 11 new patients with 5 novel homozygous variants in *PIDD1*, delineating the main phenotype associated with PIDD1-related disorder, previously described as part of large cohort studies with limited clinical information (Supplementary Table 2). First, the nonsense variant p.(Gln863*) was reported to segregate in five subjects with severe nonsydnromic ID and behavior issues [8]. Interestingly, one of them was reported to have subtle lissencephaly on the brain MRI. Shortly after, two subjects featuring moderate ID and three displaying severe ID, seizures and psychosis were reported carrying the homozygous splice site variant c.2275–1G>A most likely resulting in a frameshift of the reading frame (p.(Arg759Glyfs*1)) and the missense c.2584C>T, p.(Arg815Trp) variants, respectively [9]. Similarly, the homozygous p.(Arg637*) variant was just recently reported in a subject with mild intellectual disability and undersulcation of the bilateral temporal-frontal cortex consistent with the pachygyria/lissencephaly spectrum [10]. All affected individuals of our cohort presented with developmental delay and ID that was mostly in the mild to moderate range. Neuropsychiatric features were predominant and included self-mutilation, hetero-aggressive behavior, delusions and hallucinations. ADHD and ASD were diagnosed in half and one third of the cohort, respectively. Seizures occurring in four subjects were well controlled with anti-seizure medications.

Remarkably, all patients for whom brain MRIs were available showed a spectrum of cerebral cortical anomalies, mainly consistent with anterior-predominant pachygyria. Remarkably, this neuroradiological pattern strikingly overlaps the CRADD-related TLIS. The term lissencephaly (LIS) encompasses a spectrum of malformation of cortical development (MCD) due to mutations in genes encoding proteins of the neuronal cytoskeleton that play crucial functions in neuronal migration [13]. Current classification of LIS takes into account the severity (grade) and gradient of the gyral malformation, cortical thickness, and presence of associated brain malformations [14]. While the classical LIS refers to a smooth brain with total absence of cortical convolutions (complete LIS) and markedly thickened (10–20 mm) cortex, milder phenotypes include ‘thin’ LIS characterized by a less severely thickened (5–10 mm) cortex and pachygyria, recognizable for the presence of simplified convolutional pattern with few, broadened gyri and shallow sulci. The CRADD-related disorder belongs to this mild spectrum, accounting only for a minority of the genetic causes of LIS identified so far (≈1%) [14].

A recent study has explored the functional effect of several missense, nonsense and splice site *PIDD1* variants (p.(Arg815Trp), p.(Gln863*), and c.2275-1G>A) impacting the DD. This study showed that, while there was no apparent effect on the PIDD1 protein stability or on its autoprocessing, the variants did affect binding with CRADD, cellular localization, and Caspase-2 activation [10]. Functional assays in neuronal cells were not available, as single-cell RNAseq studies indicated that spatiotemporal overlap in expression of PIDD1 and its functional partners may be limited and highly specific [10].

Despite the lack of functional studies unveiling the impact of PIDD1 deficiency on neuronal migration, a comparison with other LIS-related disorders with similar radiological pattern is warranted and may help clinicians to recognize this novel NDD in the clinical setting. First, it must be noticed that megalencephaly is only described in the CRADD-related disorder while it is absent in PIDD1- and other LIS-related disorders. Further, seizure occurring only in a few of our patients had a very favorable course comparing to some other LIS-related disorders. Moreover, the presence of a predominant anterior gradient in association with posterior fossa anomalies in a third of our cases points to consider PIDD1-related disorder in the differential diagnosis with those affecting the Reelin signaling (i.e., *VLDLR*- and *RELN-*related disorders). However, none of them displayed the severe cerebellar hypoplasia typically observed in the Reelin disorders [15]. Of note, only three brain MRIs were available for the previous PIDD1 patients, suggesting that cortical anomalies may have been overlooked.

Similarly, the frequency of the neuropsychiatric features may have been underestimated given the lack of thorough clinical details in the previous reports [8, 9]. We in fact identified a high rate of neuropsychiatric features in our cohort, including aggressive behavior, psychosis, ADHD/Attention deficiency and ASD. The remarkable association of cortical anomalies with neuropsychiatric features is in line with current literature revealing that psychiatric symptoms may occur in up to a third of cases with MCD, including pachygyria [16, 17]. Notably, aggressive behavior has also been described in several subjects with biallelic CRADD variants [18]. A growing body of studies further supports the close relation between neuronal migration defects and neuropsychiatric disorders such as schizophrenia and ASD [19, 20], suggesting common pathomechanisms that affect the cytoskeleton architecture and dynamics leading to mis-localization of the migrating neurons and disruption of intracortical connections [21].

Five potential human PIDD1 mRNA transcript variants have been reported [6]. The longest PIDD1 mRNA transcript (NM_145886.4) encodes a protein of 910 amino acids that contains seven leucine rich repeats (LRRs), two ZU5 domains (i.e., domains present in ZO-1 and Unc5- like netrin receptors), the UPA (uncharacterized protein domain in UNC5, PIDD and ankyrins) domain and the C-terminal death domain DD [6]. Constitutive post-translational self-processing of PIDD1 gives rise to PIDD1-N (~48 kDa) and PIDD1-C (~51 kDa), which is further processed into PIDD1-CC (~37 kDa), determining the downstream signaling events. Indeed, the PIDD-C fragment mediates activation of NFκB via the recruitment of RIP1 and NEMO, while PIDD-CC causes caspase-2 activation. The latter mechanism is critically mediated by the interaction between the DDs of PIDD-CC with CRADD which stabilizes in an open conformation and in turn recruits caspase-2 to the complex via their CARD:CARD domains [3].

The nonsense variant p.(Trp447*) lying between the two ZU25 domains and the three frameshift variants p.(Gln522fs*44), p.(Gly602fs*26) and p.(Val706hisfs*30) located within the UPA domain are predicted to undergo nonsense-mediated mRNA decay acting with a likely loss-of-function mechanism. In spite of this, patient IV:1 of family 5 carrying the variant p.(Trp447*) displayed only minor cortical anomalies on the brain MRI, suggesting that additional factors may modulate the phenotype. Conversely, pachygyria along with posterior fossa anomalies and psychiatric symptoms are all features of subjects harboring the missense variants p.(Arg862Trp) and p.(Arg815Trp) in the current and previous cohort [9]. Interestingly, both missense variants are located within the DD, like the majority of CRADD variants associated with TLIS [4] that were shown to abolish CRADD’s ability to activate caspase-2, resulting in reduced neuronal apoptosis in vitro. This finding was supported by the evidence of megalencephaly and seizure in the Cradd Knockout mice, which however did not display obvious cortical defects [4]. It is worth noting that the PIDD1 knockout mice do not display major congenital anomalies albeit a careful specimen evaluation of the cortex was not performed [22].

A recent study developed at the same time of ours has shown that pathogenic variants in the PIDD1 death domain cause mis-localization of CRADD, and fail to interact with CRADD and activate caspase-2 [10], without impacting the overall expression or stability of PIDD1, or indeed the autoprocessing of the protein. Similarly, the missense variant p.(Arg862Trp) may destabilize the interaction with CRADD1 via DDs with detrimental impact on the PIDDsome-caspasis 2 activity. In spite of the lack of functional studies, we may speculate that our nonsense and frameshift PIDD1 variants could result in a reduced or absent PIDD1 product, possibly hampering the PIDDosome complex formation and in turn the activation of caspase-2. However, the mechanism through which disruption of the PIDDsome-caspasis 2 activity leads to MCD remains to be elucidated.

Caspase-dependent apoptosis is involved in several different human diseases including neurodegenerative disorders among others [23]. Specifically, caspase 2 can be activated in neurons of PIDD-null mice by nerve growth factor (NGF) deprivation or Aβ treatment inducing the formation of the RAIDD- caspase 2 complex with consequent neuronal apoptosis [24]. Moreover, caspase 2 is hyperexpressed in hippocampal neurons of subjects operated for drug refractory temporal lobe epilepsy [25] and it is required for the synaptic changes observed in the human amyloid precursor protein transgenic mice (J20) [26].

It is also worth mentioning that pathogenic variants in genes encoding proteins interacting with PIDD1 such as MADD, FADD, DNAJ, FANC1, TRIM32, PRDX1 and AIF, are also associated with several NDDs in humans [6].

In summary, we present a cohort of subjects with PIDD1-related NDD, outlining its core phenotype mostly consistent with a non-syndromic ID, several neuropsychiatric and behavioral abnormalities and a “CRADD-like” neuroimaging pattern. This finding supports previous evidence that PIDD1/CRADD/caspase-2 signaling is pivotal for normal development of the human neocortex and cognitive function. However, further studies with iPSC-derived cerebral neurons and organoids are needed to elucidate the exact mechanism leading to MCD and neuropsychiatric features. Understanding how PIDD1 deficiency affects the correct cortical development leading in turn to functional impairment of cortical brain circuits is ultimately crucial to prevent and treat associated comorbidities such as seizure and psychiatric symptoms, improving outcomes and quality of life in the affected individuals.

## Supplementary information

Supplementary information

PIDD1 variant characteristics.

Detailed genetic, clinical, and neuroradiological features of PIDD1 patients.

Craniofacial features of subjects with PIDD1 variants

## Data Availability

All variants have been deposited into LOVD database (https://databases.lovd.nl): Individual ID # 00326201, genomic variant #0000711121: https://databases.lovd.nl/shared/variants/0000711121#00016141Individual ID # 00326203, genomic variant #0000711123 https://databases.lovd.nl/shared/variants/0000711123#00016141Individual ID # 00326204, genomic variant #0000711124 https://databases.lovd.nl/shared/variants/0000711124#00016141 Individual ID # 00326205, genomic variant #0000711125 https://databases.lovd.nl/shared/variants/0000711125#00016141 Individual ID # 00326202, genomic variant # 0000711122 https://databases.lovd.nl/shared/variants/0000711122#00016141 Individual ID # 00326200, genomic variant #0000711120 https://databases.lovd.nl/shared/variants/0000711120#00016141 The raw data of the current study are available from the corresponding author on reasonable request.
