# Supplementary material for: Pathogenic variants in PIDD1 lead to an autosomal recessive neurodevelopmental disorder with pachygyria and psychiatric features
Source: Eur J Hum Genet. 2021 Jun 24;29(8):1226–34. doi: 10.1038/s41431-021-00910-0 (PMC8385073; doi:10.1038/s41431-021-00910-0)
Supplement: Supplementary file 1 — Supplementary information [file 41431_2021_910_MOESM1_ESM.docx]

**Supplementary information**

**Supplementary table 1.** PIDD1 variant characteristics.

**Supplementary table 2.** Detailed genetic, clinical, and neuroradiological features of PIDD1 patients.

**Supplementary figure 1.** Craniofacial features of subjects with PIDD1 variants

Clinical features of patients with homozygous PIDD1 variants showing subtle and non-specific dysmorphic features such as prominent forehead in most cases; additional features include wide-spaced eyes, epicanthal folds, almond shaped eyes, thin upper lip in subject IV:1 of family 5; sparse eye brows, deep-set eyes, broad nasal root, enlarged naris, deep philtrum, full lips, broad chin in subject IV:3 of family 1; thick eyebrows, enlarged ale naris, broad chin broad are common features in affected members of family 4; subjects of family 2 display long face, downslanting palpebral fissures, short philtrum, thick lower lips and broad chin; individuals of family 3 presents with sparse lateral eyebrows and short phlitrum; patient IV:3 of family Manipal-1 and the individual III-4 of the family AS105 III-4, both previously reported do not display obvious dysmorphism.
