# Supplementary material for: Pathogenic variants in PIDD1 lead to an autosomal recessive neurodevelopmental disorder with pachygyria and psychiatric features
Source: Eur J Hum Genet. 2021 Jun 24;29(8):1226–34. doi: 10.1038/s41431-021-00910-0 (PMC8385073; doi:10.1038/s41431-021-00910-0)
Supplement: Supplementary file 2 — PIDD1 variant characteristics. [file 41431_2021_910_MOESM2_ESM.docx]

| **Family ID** | **1** | **2** | **3** | **4** | **5** | **6** |  |
| --- | --- | --- | --- | --- | --- | --- | --- |
| **Patient ID** | **IV:3** | **IV:3, IV:4** | **IV:1, IV:3** | **IV:2, IV:3, IV:4** | **IV:1** | **IV:1, IV:2** |  |
| **Variant annotation** | NC_000011.10  GRCh38/hg38 position  (DNA change) | 11:g.799456G>A | | 11:g.801587C>T | 11:g.800373 GTCACdel | 11:g.801283TGdel | 11:g.800874  CCdel |
|  | cDNA change ( NM_145886.4) | c.2584C>T | | c.1340G>A | c.2116_2120del | c.1564_1565del | c.1804_1805del |
|  | Protein change ( NP_665893.2) | p.(Arg862Trp) | | p.(Trp447*) | p.(Val706Hisfs*30) | p.(Gln522Glufs*44) | p.(Gly602fs*26) |
|  | Zygosity | Hom | | Hom | Hom | Hom | Hom |
|  | dbSNP ID | rs747620551 | | - | - | - |  |
|  | gnomAD v3  (highest subpopulation) | 0.00001247,3/273,720  0.00002780, 3/107906  (Euepean-non-finish) | | absnet | absent | absent | absent |
| **Allele frequencies (PM2)** | gnomAD v2.1.1  (highest subpopulation) | 0.00001971 3/152174 0.00004826 2/414468  (African/African-American) | | absnet | absent | absent | absent |
|  | Frequency in ensembl browser | absent | | absent | absent | absent | absent |
|  | Iranome | absent | | absent | absent | absent | absent |
|  | GME Variome | absent | | absent | absent | absent | absent |
|  | 100.000 Genome project | absent | | absent | absent | absent | absent |
|  | Centogene | absent | | absent | absent | 1(hmz) | 1(hmz, 2htz) |
|  | UK Biobank | absent | | absent | absent | absent | absent |
|  | in-house database | absent | | absent | absent | absent | absent |
|  | GERP | 4.74 | | 2.9 | - | - |  |
| ***In silico* predictions (PP3)** | CADD | 31 | | 50 | - | - | - |
|  | Polyphen-2 | Probably damaging | |  |  | - |  |
|  | SIFT | Damaging | |  |  | - |  |
|  | Provean | Damaging | |  |  | - |  |
|  | MutationTaster | Disease causing | | Disease causing | Disease causing | Disease causing | Disease causing |
| **ACMG classification** |  | Likely pathogenic (PM2,PP3,PP1,PP4) | | Pathogenic (PVS1,PM2,PP1  PP3) | Pathogenic  (PVS1,PM2,PP1) | Likely pathogenic  (PVS1,PM2) | Likely pathogenic  (PVS1,PM2) |

Reference sequence: NM_004269

**Supplemental table 1:*PIDD1* variant characteristics**

Legend ACMG criteria:

PVS1 Null variant (nonsense, frameshift, canonical ±1 or 2 splice sites, initiation codon, single or multiexon deletion) in a gene where LOF is a known mechanism of disease;

PM2 Absent from controls (or at extremely low frequency if recessive) in Exome Sequencing Project, 1000 Genomes Project, or Exome Aggregation Consortium; PP1 Cosegregation with disease in multiple affected family members in a gene definitively known to cause the disease; PP3 Multiple lines of computational evidence support a deleterious effect on the gene or gene product (conservation, evolutionary, splicing impact, etc.); PP4 patient’s phenotype or family history is highly specific for a disease with a single genetic etiology.
