# Supplementary figures and images for: Pathogenic variants in PIDD1 lead to an autosomal recessive neurodevelopmental disorder with pachygyria and psychiatric features
Source: Eur J Hum Genet. 2021 Jun 24;29(8):1226–34. doi: 10.1038/s41431-021-00910-0 (PMC8385073; doi:10.1038/s41431-021-00910-0)

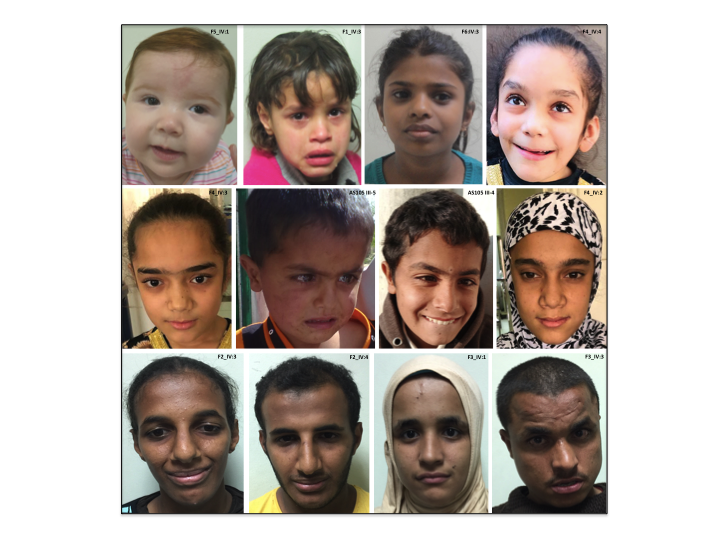

Supplement: Supplementary file 4 — Craniofacial features of subjects with PIDD1 variants [file 41431_2021_910_MOESM4_ESM.tif]
